# Genetic analysis of dTSPO, an outer mitochondrial membrane protein, reveals its functions in apoptosis, longevity, and Aβ42-induced neurodegeneration

**DOI:** 10.1111/acel.12200

**Published:** 2014-02-21

**Authors:** Ran Lin, Alessia Angelin, Federico Da Settimo, Claudia Martini, Sabrina Taliani, Shigong Zhu, Douglas C Wallace

**Affiliations:** 1 Department of Physiology and Pathophysiology, School of Basic Medical Sciences, Health Science Center, Peking UniversityBeijing, 100191, China; 2 Center for Mitochondrial and Epigenomic Medicine, Children’s Hospital of Philadelphia Research InstitutePhiladelphia, PA, 19104, USA; 3 Dipartimento di Farmacia, Università di Pisavia Bonanno 6, 56126, Pisa, Italy; 4 Department of Pathology and Laboratory Medicine, Perelman School of Medicine, University of PennsylvaniaPhiladelphia, PA, 19104, USA

**Keywords:** apoptosis, Drosophila, longevity, mitochondria, neurodegeneration, TSPO

## Abstract

The outer mitochondrial membrane (OMM) protein, the translocator protein 18 kDa (TSPO), formerly named the peripheral benzodiazepine receptor (PBR), has been proposed to participate in the pathogenesis of neurodegenerative diseases. To clarify the TSPO function, we identified the *Drosophila* homolog, CG2789/dTSPO, and studied the effects of its inactivation by P-element insertion, RNAi knockdown, and inhibition by ligands (PK11195, Ro5-4864). Inhibition of dTSPO inhibited wing disk apoptosis in response to γ-irradiation or H_2_O_2_ exposure, as well as extended male fly lifespan and inhibited Aβ42-induced neurodegeneration in association with decreased caspase activation. Therefore, dTSPO is an essential mediator of apoptosis in *Drosophila* and plays a central role in controlling longevity and neurodegenerative disease, making it a promising drug target.

## Introduction

The translocator protein 18 kDa (TSPO), formerly named peripheral benzodiazepine receptor (PBR), is a low-molecular-weight protein localized to the outer mitochondrial membrane (OMM), encompassing five transmembrane hydrophobic domains (Papadopoulos *et al*., 2006). In mammals, TSPO is expressed across tissues with the highest expression observed in steroid synthesizing tissues (Lacapere & Papadopoulos, 2003). In addition to steroid biosynthesis (Midzak *et al*., 2011), it has been implicated in heme biosynthesis (Taketani *et al*., 1994), calcium signaling (Hong *et al*., 2006), protein import (Hauet *et al*., 2005), cell proliferation and differentiation (Galiegue *et al*., 2004; Rechichi *et al*., 2008), cell apoptosis (Ritsner *et al*., 2003; Rechichi *et al*., 2008; Zeno *et al*., 2009), and mitochondrial oxidative phosphorylation (OXPHOS) (Larcher *et al*., 1989). Mammalian TSPO has also been proposed to be the central outer membrane polypeptide in the mitochondrial permeability transition pore (mPTP) (Ricchelli *et al*., 2011; Sileikyte *et al*., 2011).

Translocator protein 18 kDa is of particular interest to neurodegeneration, being abundantly expressed in glial cells recruited and activated during neuro-inflammation. Thus, TSPO intensity is increased in Alzheimer’s disease (AD), stroke, and multiple sclerosis (MS), making imaging TSPO ligands an important system for diagnosing neurodegenerative diseases (Venneti *et al*., 2006; Lavisse *et al*., 2012). A single TSPO amino acid substitution (Ala147Thr) has been associated with human adult separation anxiety (Costa *et al*., 2009) and TSPO expression in neurons has been implicated in modulating long-term potentiation and learning (Tokuda *et al*., 2010). Hence, TSPO may be an important target for treating neurological diseases (Veiga *et al*., 2007). TSPO has also been implicated in the pathogenesis of heart disease (Bird *et al*., 2010; Schaller *et al*., 2010), atherosclerosis (Bird *et al*., 2010), and inflammatory bowel disease (Ostuni *et al*., 2010). In addition, TSPO expression is increased in a variety of cancers. Consequently, TSPO ligands have been reported to have therapeutic effects on certain tumors through modulation of cellular proliferation and apoptosis (Furre *et al*., 2005; Fafalios *et al*., 2009; Zheng *et al*., 2011).

Although TSPO ligands have been widely applied in clinical imaging and therapeutics, the function of TSPO in the biology of the cell and mitochondrion are still poorly understood. To address this deficiency, we have analyzed the physiological consequences of inactivation of the *tspo* gene in *Drosophila*. Analysis of dTSPO-deficient *Drosophila* has confirmed that dTSPO plays a central role in the regulation of apoptosis and that its modulation can extend lifespan and ameliorate the toxicity of Aβ42 over-expression.

## Results

### Drosophila has a TSPO which can be inactivated

**Figure 1:**
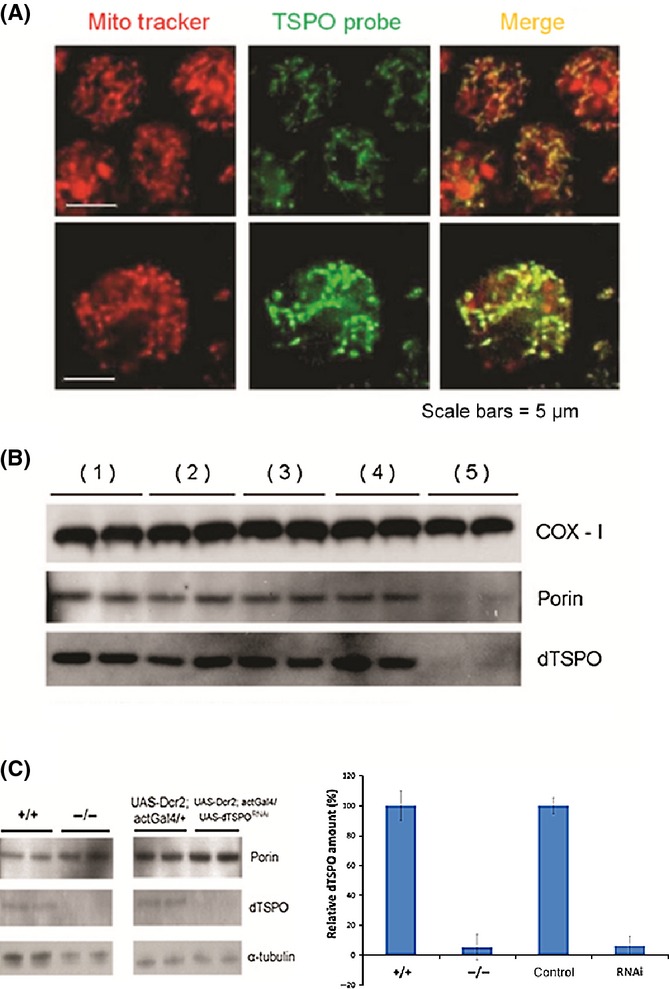
The localization of dTSPO and its depletion in *tspo* −/− and dsRNA knockdown flies. (A) The mitochondrial localization of dTSPO in S2 cells. S2 cells were stained with MitoTracker Deep Red (red) and TSPO fluorescent probe (green) sequentially. (B) Western blot of dTSPO, COX-I (IMM), and Porin (VDAC) (OMM) in isolated mitochondria from whole bodies of wild type flies treated with increasing concentrations of digitonin which dissolves the OMM releasing its proteins. dTSPO is lost along with porin. For (1)–(5), the concentrations of digitonin are 0, 0.25, 0.5, 1, 2 mg mL^−1^. (C) Western blot showing depletion of dTSPO in *tspo* −/− and dsRNA (RNAi) whole-body knockdown flies (mixture of equal numbers of male and female flies). Porin (VDAC) provides the OMM mitochondrial control and α-tubulin provides the total protein loading control. The densitometry of western films was shown (*N* = 2). Bars report mean ± SEM.

The *tspo* gene is highly conserved from bacteria to humans. Therefore, we were able to identify the *Drosophila tspo* homologue, CG2789/dTSPO, using a BLAST search of the *Drosophila* protein sequences for homology with the human TSPO protein sequence. The alignment of the *Drosophila* TSPO polypeptide with those of human and other species is shown in Fig. S1. The mitochondrial localization of the dTSPO protein was confirmed by the co-localization of MitoTracker Deep Red which is resistant to fixation in cultured *Drosophila* S2 cells together with the synthetic fluorescent TSPO affinity probe N-(5-Isothiocyanato-2-phenylindol-3-ylglyoxyl)-N′-(7-nitrobenz-2-oxa-1,3-diazol-4-yl)-1,6-diaminohexane (named compound 18) (Taliani *et al*., 2010) (Fig. 1A).

Disruption of the *Drosophila* OMM with digitonin removed Voltage-dependent anion channel (VDAC, also named porin), the marker of OMM, together with dTSPO. The inner mitochondrial membrane (IMM) localized cytochrome c oxidase subunit I (COX-I) protein was not affected. These results confirm the OMM location of dTSPO (Fig. 1B).

A review of the *Drosophila* mutant repository revealed a strain in which a P-element had inserted into the *tspo* gene, *tspo*[EY00814]. We also depleted the *Drosophila* TSPO by expression of dsRNA (RNAi) homologous to the dTSPO mRNA regulated by the Gal4/UAS system in various fly tissues. Western blot analysis confirmed that the *tspo* P-element inactivation (*tspo* −/−) and whole body dsRNA dTSPO knockdown flies lacked dTSPO protein, while another OMM protein, porin (VDAC), was unchanged (Fig. 1C). Still, both *tspo* −/− mutant flies and dsRNA whole body knockdown flies were viable and grossly normal and had comparable developmental timing as wild type flies (Fig. S2).

### Inactivation of dTSPO protects cells from apoptosis

**Figure 2:**
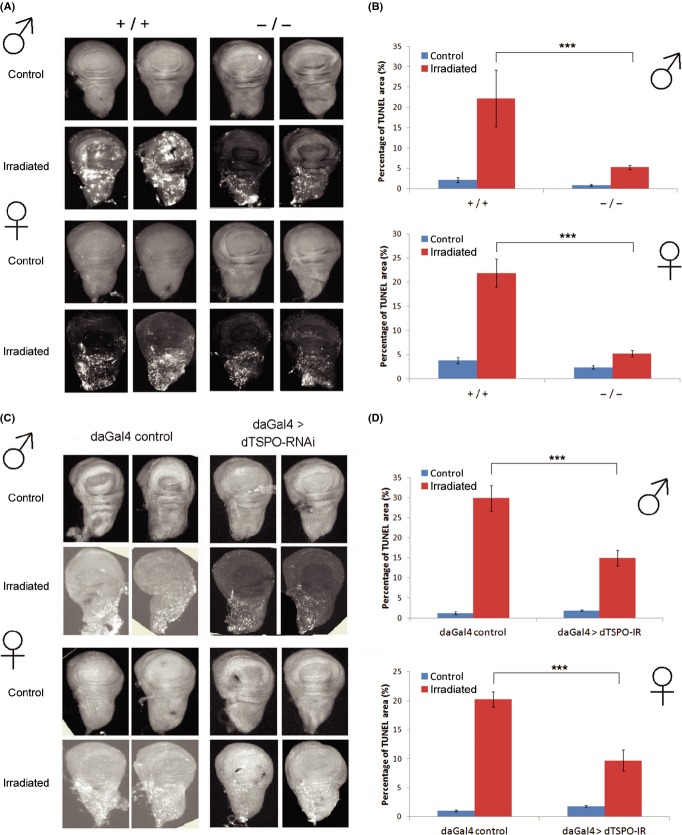
Inhibition of apoptosis in wing disks by dTSPO inactivation. (A) Wing disc apoptosis of male (upper) and female (lower) 3rd-instar larvae of wild type or *tspo* −/− flies irradiated with 30 Gray of γ-ray and TUNEL stained. (B) Quantification of apoptosis in (A) by measuring the area of the TUNEL positive pixels divided by total disk pixels, *n* = 3 to 10 wing disks quantified. (C) and (D) The same method but comparing control versus dTSPO dsRNA knockdown flies, *n* = 6–9. Bars report mean ± SEM. ***P < 0.001.

**Figure 3:**
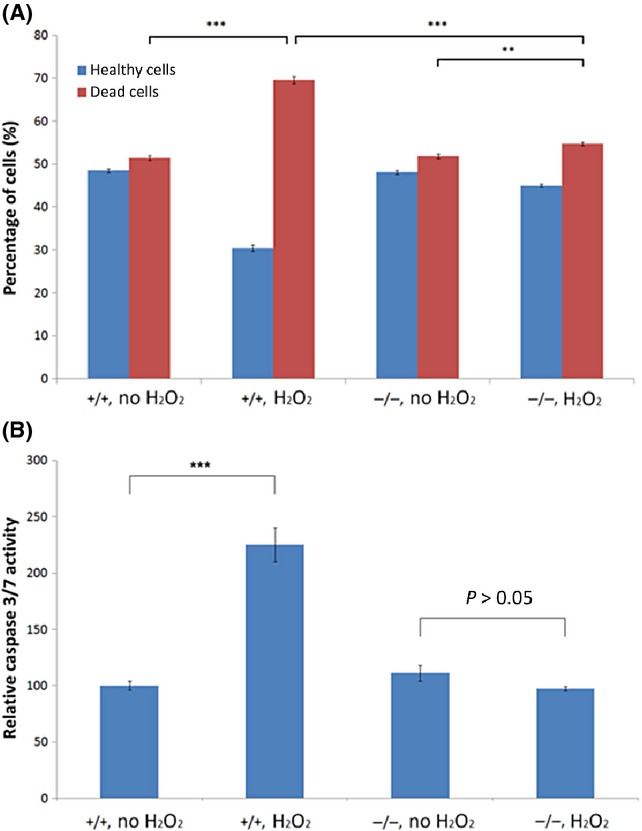
Inhibition of apoptosis in isolated larval brain cells by dTSPO inactivation. Effect of H_2_O_2_ on induction of apoptosis in 3rd-larvae brain cells from *tsps* +/+ or *tspo* −/− flies (mixture of equal numbers of male and female flies). (A) Flow cytometry quantification of FITC-Annexin V positive cells (dead cells) versus FITC-Annexin V negative and Propidium Iodide negative cells (healthy) cells (each bar, *n* = 3–6). (B) Assay of caspase 3/7 activity (*n* = 3). Bars report mean ± SEM. *P < 0.05, **P < 0.01, ***P < 0.001.

If dTSPO participates in the intrinsic pathway of apoptosis in *Drosophila*, its inactivation should inhibit cell death. Using irradiation-induced apoptosis in *Drosophila* 3rd-instar larvae tissues (Wichmann *et al*., 2006) (Fig. 2), we found that γ-ray at 30 Gray followed by 3 h recovery stimulated apoptosis in wing disc cells detected by TUNEL staining (Fig. 2A,C). Quantification of the ratio of area of TUNEL positive pixels versus whole wing disc pixels revealed that apoptosis induction was drastically suppressed in *tspo* −/− and whole-body dTSPO knockdown flies, both male and female (Fig. 2B,D). Similarly, cells isolated from 3rd-instar larval brains exposed to H_2_O_2_ and monitored for apoptosis by Propidium Iodide (PI)/FITC-Annexin V double staining and flow cytometry (Fig. 3A) as well as by caspase 3/7 activity (Fig. 3B) revealed significant suppression of apoptosis in *tspo* −/− fly cells. Therefore, inactivation of dTSPO inhibited apoptosis in flies of both genders.

### Inactivation of dTSPO extends lifespan

**Figure 4:**
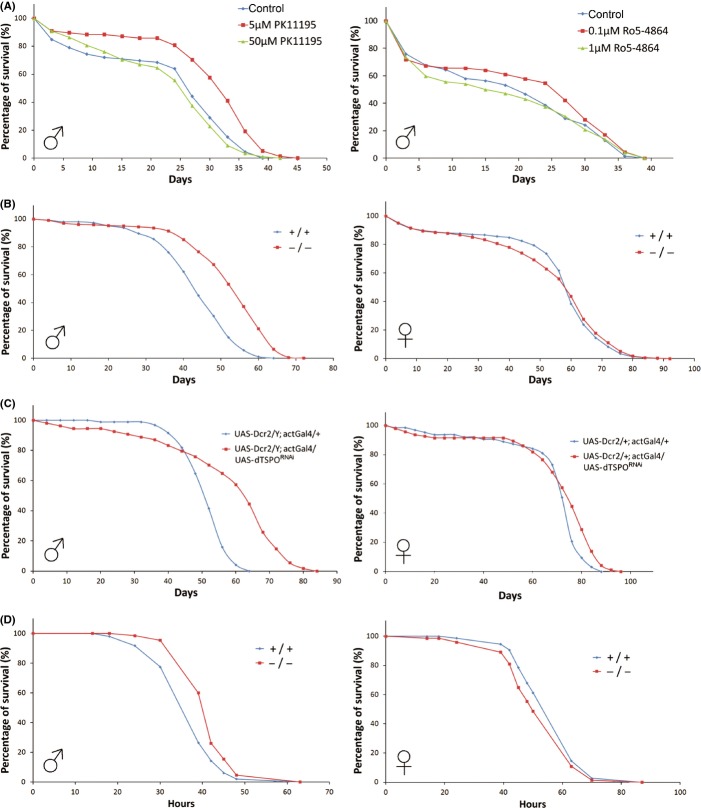
Effects of dTSPO inhibition on *Drosophila* lifespan and oxidative stress resistance. (A) Left, exposure of wild type (*tspo* +/+) male flies to 5 or 50 μm PK11195 (*n* = 78–88 counted for each group). Right, exposure of *tspo* +/+ flies to 0.1 or 1 μm Ro5-4864 (*n* = 62–72 counted for each group). Drugs administered in 4% sucrose at 25 °C. (B) Extension of lifespan of *tspo* −/− males (left) though not female (right) relative to *tspo* +/+ flies maintained in standard cornmeal medium at 25 °C, *n* = 250–350.(C) Extension of lifespan of whole body dTSPO dsRNA knockdown males (left) though not female (right) relative to control flies maintained on cornmeal medium at 25 °C, *n* = 50–100. (D) Protection of males (left) but not females (right) *tspo* −/− flies to exposure to 5% H_2_O_2_ in 5% sucrose/PBS at 25 °C relative to *tspo* +/+ flies, *n* = 50–75.

If apoptosis in *Drosophila* influences longevity as implicated in mammals (Biteau *et al*., 2011; Raffaello & Rizzuto, 2011; Rufini *et al*., 2013), loss of dTSPO should extend lifespan, and treatment of male flies with the TSPO antagonist ligand should extend lifespan. Treatment with PK11195 (left) did extend lifespan at moderate concentrations. However, at higher concentration, lifespan was unchanged. Treatment with another TSPO antagonist ligand, Ro5-4864 (right), exerted similar though milder effect on lifespan (Fig. 4A, Table S1). Genetic depletion of dTSPO in either *tspo* −/− homozygous mutant (Fig. 4B, Table S1) or whole body knockdown flies (Fig. 4C, Table S1) also extended lifespan, however, the effect was male-specific.

As resistance to various stresses is frequently associated with longevity, the sensitivity to oxidative stress induced by H_2_O_2_ and metabolic stress induced by starvation were analyzed in wild type and *tspo* −/− flies. Male *tspo* −/− flies were more resistant to H_2_O_2_ than wild-type flies, though female flies were not (Fig. 4D) (With log rank test for male, *P* = 0.0015; for female, *P* = 0.0932). dTSPO deletion did not affect sensitivity to starvation and heat stress, in neither male nor female flies (Fig. S3).

To determine the relation of dTSPO expression to aging, we analyzed the dTSPO mRNA and protein levels at 22 °C throughout adult life. The dTSPO mRNA increased continuously from 0 to 30 days after eclosion (DAE) (Fig. S4A). This was paralleled by a progressive increase in dTSPO protein from 30 to 60 DAE. Interestingly, dTSPO protein levels were elevated immediately after eclosion, suggesting that dTSPO may be carried over from the larval or pupal stage (Fig. S4B).

### Inactivation of dTSPO imparts resistance to neurodegenerative disease

**Figure 5:**
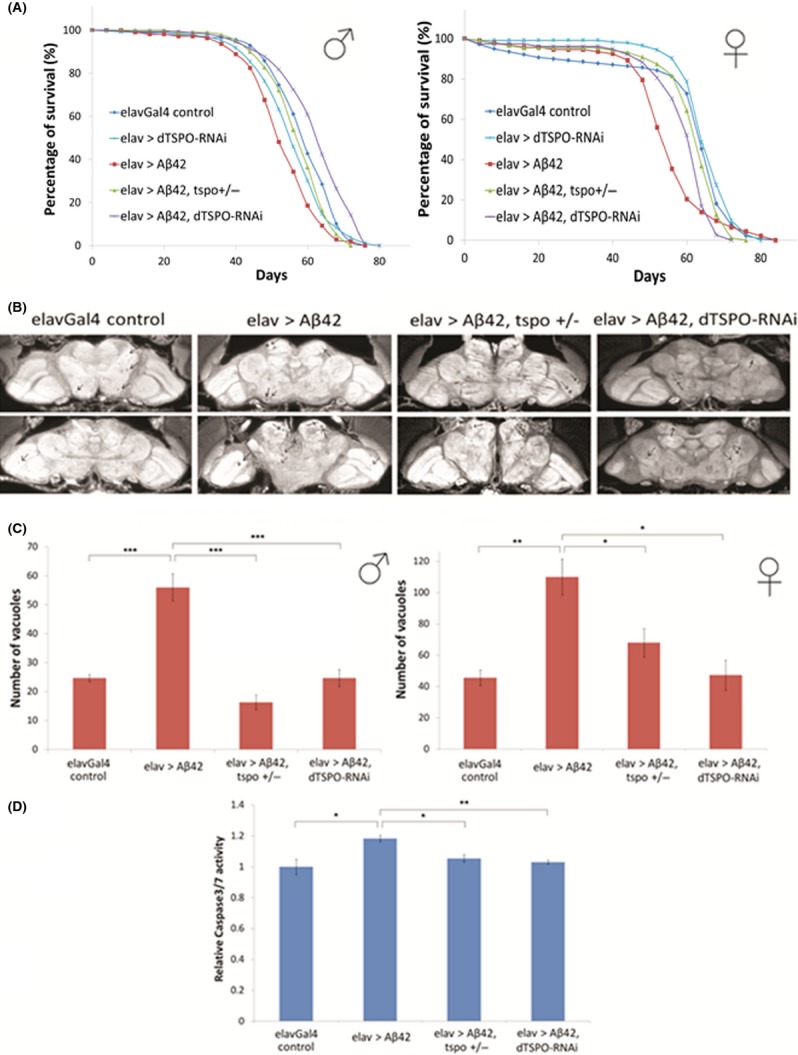
Effect of dTSPO reduction on Aβ42-induced neurodegeneration. Systemic reduction (*tspo* +/−) or neuron-specific knockdown of dTSPO restored neuronal human Aβ42-induced lifespan reduction in male and female flies. (A) Survival curves of male (left) and female (right) flies in response to neuronal expression of human Aβ42 (elav>Aβ42) relative to control flies (elavGal4 control) lacking the UAS-Aβ42 target gene and amelioration of the lifespan reduction induced by Aβ42 expression by partial systemic inactivation of *tspo* +/− or neuronal dsRNA inactivation of dTSPO expression (elav>Aβ42, dTSPO-RNAi). (males, *n* ≈ 200 and females, *n* ≈ 100). (B, C) Reduced neuronal human Aβ42 induced tissue loss in response to systemic (*tspo* +/−) or neuronal depletion (dTSPO-RNAi). (B) Representative histological sections for only male brains were displayed. Arrows indicate regions of neuronal loss (vacuole-like), DAE = 45. (C) Quantification of number of vacuole-like regions per single head of male (left, *n* = 3–6 for each genotype, DAE = 45) or female (right, *n* = 3–4 for each genotype, DAE = 60) flies. (D) Effect of dTSPO depletion on neuronal Aβ42-induced male fly head caspase 3/7 activation (*n* = 3), DAE = 20. Each bar reports mean ± SEM. *P < 0.05, **P < 0.01, ***P < 0.001.

As aging is the leading risk factor for AD and apoptosis of neurons has been reported in AD (Castro *et al*., 2010), we investigated whether inactivation of dTSPO could ameliorate the toxic effects of over-expression of the human AD-associated amyloid peptide, Aβ42, transcribed from the neuron-specific promoter (elav > Aβ42). Aβ42 expression in the fly brain induces neurodegeneration, shortens lifespan (Fig. 5A, Table S2), and results in the disintegration of the brain tissue (Iijima *et al*., 2004). Both the systemic partial reduction of brain dTSPO in *tspo* +/− flies (elav>Aβ42, tspo +/−) and the neuronal-specific dsRNA knockdown of dTSPO (elav>Aβ42, dTSPO-RNAi) reduced the Aβ42-induced toxicity and restored the normal lifespan (Fig. 5A, Table S2), even though knockdown of dTSPO in neurons (elav>dTSPO-RNAi versus elavGal4 control) did not extend lifespan of wild type flies (Fig. 5A, Table S2). Despite the male-specific enhanced longevity and oxidative stress resistance in dTSPO-depleted flies, the depletion of dTSPO on Aβ42-expressing flies was comparably protective for both male and female flies.

To determine the physical basis for the protective effect of dTSPO depletion on Aβ42-toxicity, the tissue integrity was monitored using the number of vacuole-like lesions in brain slices of aged flies. Both partial systemic inactivation (*tspo* +/−) and neuronal knockdown of dTSPO reduced the numbers of brain lesions (Fig. 5B,C).

To assess why Aβ42-induced brain vacuolation was reduced by dTSPO depletion, we determined the neuronal activity of caspase 3/7 in Aβ42 over-expressing flies, caspase activation being associated with Aβ42 toxicity (Castro *et al*., 2010; Rohn, 2010). While neuronal expression of human Aβ42 increased caspase 3/7 activity by 20% in fly heads by 20 DAE, systemic partial inactivation of dTSPO (*tspo* +/−) and neuron-specific knockdown of dTSPO returned the caspase activity to that of normal brain tissue (Fig. 5D).

To determine whether dTSPO depletion reduced Aβ42 toxicity by inhibiting Aβ42 expression, the level of Aβ42 mRNA was monitored by quantitative RT-PCR. In Aβ42 over-expressing flies, inactivation of dTSPO did not diminish Aβ42 mRNA levels in neurons. Hence, the rescue effects of dTSPO depletion are not caused by altered Gal4/UAS system or altered synthesis/stability of Aβ42 mRNA (Fig. S5).

### The dTSPO is necessary to sustain mitochondrial function

**Figure 6:**
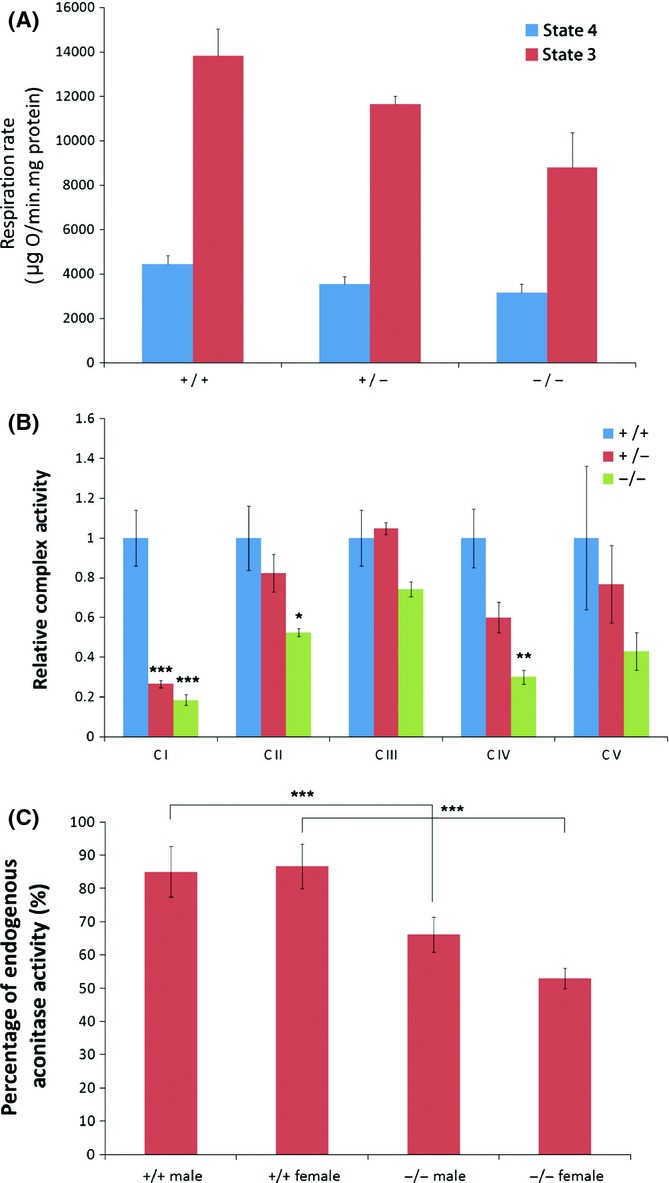
Effect of dTSPO depletion on 7–10 DAE *Drosophila* mitochondrial OXPHOS. Mitochondria were isolated from whole body homogenates of *tspo* +/+, *tspo* +/−, and *tspo* −/− flies (mixture of equal number of males and females). (A) Mitochondrial oxygen consumption rate when metabolizing site I (pyruvate + malate) substrates in the absence (state 4) or presence (State 3) of ADP. *N* = 3 for each genotype. (B) Mitochondrial OXPHOS complexes I to V specific activities normalized using citrate synthase activity. *N* = 3 for each enzyme value. (C) Mitochondrial aconitase activity in male and female *tspo* +/+ versus −/− flies expressed as the percentage ratio of the endogenous activity divided by the Fe^2+^-recovered activity. *N* = 3 for each assay. *P < 0.05, ** *P* < 0.01, *** *P* < 0.001.

To determine whether dTSPO was important in sustaining mitochondrial function, mitochondrial respiration, OXPHOS complex specific activities, or mitochondrial ATP production and mitochondrial aconitase inactivation these parameters were assessed at 2–5 DAE and 7–10 DAE flies. While the 2–5 DAE flies showed minimal changes in OXPHOS (Fig. S5), the 7–10 DAE flies exhibited reduced mitochondrial respiration, OXPHOS enzyme activities, and increased mitochondrial oxidative stress (Fig. 6).

Mitochondria isolated from flies at 2–5 DAE had a basal respiration rate using site I substrates (pyruvate and malate) that was mildly increased in the absence of ADP (state IV), but the ADP-stimulated respiration rate (state III) was unaffected (Fig. S6A). This resulted in a modest decreased Respiration Control Ratio (RCR) (state III/state IV respiration rate) in association with a slightly reduced membrane potential (Fig. S6B). The specific activities of the electron transport chain complexes I, II, III and IV were not significantly reduced (Fig. S6C) nor was mitochondrial oxidative stress (Fig. S6D) significantly increased in *tspo* +/− or −/− 2–5 DAE flies.

By contrast, mitochondria isolated from 7 to 10 DAE dTSPO-deficient (*tspo* −/−) flies resulted in a reduction in both basal and ADP-stimulated respiration using site I substrates, resulting in a constant RCR (Fig. 6A). This was associated with a striking reduction in complexes I specific activity in both the *tspo* +/− and −/− flies and substantial reductions in complex II and IV activities in *tspo* −*/*− flies (Fig. 6B). Despite this obvious reduction of OXPHOS, the *tspo* +/− and −/− flies did not have reduced ATP levels relative to controls (Fig. S7). Finally, the 7–10 DAE *tspo* −/− *Drosophila* exhibited a marked reduction in mitochondrial aconitase activity revealing chronic mitochondrial oxidative damage (Fig. 6C), though the level of malondialdehyde (MDA), a by-product of lipid peroxidation measured in TBARS assay (thiobarbituric acid reactive substances), in older male and female mutant flies was not significantly increased relative to wild-type flies. Hence, the increased mitochondrial ROS seen in mutant flies did not increase ROS-generated macromolecular damage (Fig. S8).

## Discussion

The TSPO has been proposed as an important OMM factor for controlling apoptotic cell death. In mammals, there are 2 *tspo* gene paralogs, *tspo1* and *tspo2*, having slightly different functions (Fan *et al*., 2009). In *Drosophila*, by contrast, we have found only one *tspo* gene (CG2789/dTSPO). Therefore, unlike mammalian systems, inactivation of the *Drosophila tspo* gene has permitted direct examination of TSPO function. This has revealed that dTSPO inactivation in *Drosophila* inhibits apoptosis and mitochondrial bioenergetics while ameliorating neurodegenerative disease and extending lifespan.

Genetic inactivation of dTSPO reduced apoptotic cell death triggered by γ-ray in living animals and by chemical inducers (H_2_O_2_) in isolated brain cells. Hence, our results confirmed that *Drosophila* mitochondria still play an essential role in apoptosis and that this is mediated by the dTSPO located in the OMM.

While apoptosis plays a significant role in development in many species, depletion of dTSPO did not affect the development or gross morphology of *Drosophila*. This suggests that the signals and/or mechanisms of normal developmental apoptosis and stress-induced apoptosis may be different in flies.

Systemic inhibition of dTSPO in *Drosophila*, either by pharmacological intervention (PK11195, Ro5-4864 exposure) or by genetic inactivation, resulted in a significant extension of lifespan in male flies. Genetic inactivation of dTSPO also rendered male flies resistant to H_2_O_2_.

The systemic knockdown of dTSPO extended lifespan even in the face of the severe inhibition of the OXPHOS enzymes and chronically elevated mitochondrial oxidative stress. This places mitochondrial dTSPO-mediated cell death as essential for translating mitochondrial dysfunction into reduced longevity. In mammals, apoptosis can be initiated by activation of the mPTP through oxidative stress (Wallace & Fan, 2009). As mammalian apoptosis has been implicated in regulating longevity (Biteau *et al*., 2011; Raffaello & Rizzuto, 2011; Rufini *et al*., 2013), loss of cells due to the activation of the intrinsic pathway of apoptosis mediated through TSPO would seem to be central to longevity. The fact that *Drosophila* lifespan was not extended by the neuronal-specific knockdown of dTSPO in the presence of increased mitochondrial oxidative stress, but lifespan was extended with systemic inactivation of dTSPO suggests that the dTSPO knockdown effect is not neuron-specific. This contrasts with reports that over-expression of the antioxidant enzyme Cu/Zn superoxide dismutase (*Sod1*) in *Drosophila* motor-neurons does extend lifespan (Parkes *et al*., 1998).

Neuronally expressed human Aβ42 shortened the lifespan of *Drosophila* in association with increased brain cell apoptosis and both of these effects of Aβ42 were ameliorated by dTSPO deficiency in both male and female flies. Therefore, TSPO-associated apoptosis is an important factor in mediating neuronal cell death and thus neurodegenerative disease, even though neuronal dTSPO deficiency did not extend lifespan.

The differential effect of oxidative stress on male versus female flies indicates that sex differences modulate the sensitivity to oxidative stress and aging. This may be analogous to our observation that in mammalian cells a portion of the estrogen receptor protein is located in the mitochondrial matrix and when activated by 17β-estradiol rapidly increases the specific activity of mitochondrial Mn superoxide dismutase (Pedram *et al*., 2006). It is possible that similar sex-specific factors may mitigate some of the deleterious consequences of oxidative stress in females. However, these sex-specific factors may not exist in *Drosophila* brain, as the Aβ42 neurotoxicity was comparably restored by dTSPO depletion in both males and females.

While it has been reported that TSPO probes colocalized with glial markers and not neuronal markers (Venneti *et al*., 2008), the protection for Aβ42-mediated neurodegeneration when dTSPO was specifically knockeddown in fly neurons, argues that dTSPO is both expressed in neurons and important for their integrity. Our data thus support the observation that mammalian TSPO functions in hippocampal neurons and affects long-term potentiation and learning (Tokuda *et al*., 2010).

If the mPTP is involved in mediating the intrinsic pathway of apoptosis in *Drosophila*, as it is in vertebrates (Li *et al*., 2007), then our data imply that the TSPO may be an important OMM component of the mPTP. If this is the case, then it may be interacting with ATP synthase dimers that have recently been proposed to form the IMM component of the mPTP (Giorgio *et al*., 2013). In *Drosophila*, the Ca^2+^ efflux mediated by the fly mPTP counterpart is associated with tetracaine- and thiol-sensitive IMM depolarization but is not modulated by Ca^2+^ via cyclophilin D nor is it associated with mitochondrial swelling or cytochrome c release (von Stockum *et al*., 2011). Hence, the mechanism by which dTSPO depletion is coupled to inhibition of apoptosis still requires further clarification.

Similarly, the mechanism by which dTSPO depletion imparts protection from neurodegenerative disease and extension of lifespan in the face of OXPHOS dysfunction merits further investigation. Under the conventional view of the mitochondrial free-radical theory of aging, aging is caused by damage to macromolecules by mitochondrial dysfunction-triggered ROS over-production. However, this ‘theory’ remains contested due to observations such as those in this manuscript where mitochondrial aconitase inactivation suggested increased mitochondrial ROS production yet lifespan was extended and MDA levels were not increased. Possibly, the increased mitochondrial ROS production resulting from dTSPO depletion activated antioxidant defences mitigating macromolecular damage through induction of hormesis via JNK pathway and thus extended lifespan. In *Drosophila,* the JNK pathway has been shown to be activated by ROS and activated JNK can extend lifespan (Biteau *et al*., 2011). While JNK can also sensitize cells to apoptosis, this effect might be blocked by the dTSPO deficiency. The net result would be protection from neurodegenerative disease and extension of lifespan.

In summary, we have found that dTSPO is an OMM protein that is essential for *Drosophila* apoptosis and that elimination of dTSPO inhibits apoptosis, inhibits neurodegeneration, and extends lifespan. Given the high species conservation of TSPO, the role of TSPO in apoptosis must be both ancient and conserved. Hence, pharmacological modulation of TSPO may be a productive approach for treating degenerative diseases.

## Experimental procedures

### Production of dTSPO polyclonal antibody

A 15 amino acid peptide spanning from N164 to S178 of CG2789/dTSPO (NH_2_-CNPEKEQAPKDEEKPS-COOH) was used to produce a *Drosophila* polyclonal antibody. Peptide synthesis, conjugation, antiserum production, ELISA screening and affinity purification were done by Covance, Inc. (Princeton, NJ, USA).

### Fly stocks

*Drosophila* were raised on standard cornmeal medium at 22 °C (used for qRT-PCR and western blot of dTSPO in aged flies) or 25 °C (used in all other assays). The *tspo*[EY00814] strain, obtained from the Bloomington *Drosophila* Stock Center, has a P-element insertion in the 3′ regulatory region of *tspo* gene. The UAS-dTSPO-RNAi stock was obtained from Vienna *Drosophila* RNAi Center (VDRC) and contained a transgene which can be transcribed into a dsRNA that targets the dTSPO mRNA. Pan-neuronal driver elav-Gal4 and UAS-human WT Aβ42 were kindly provided by Dr. Yi Zhong in Cold Spring Harbor Laboratory and Tsinghua University, China. The drivers of UAS-Dcr2; actin-Gal4/CyO, and da-Gal4 were also obtained from Bloomington. The strains were all backcrossed to w^1118^ background.

### S2 cell culture and staining

*Drosophila* Schneider’s cells (S2 cells) were maintained in Schneider’s *Drosophila* Medium (Gibco, Grand Island, NY, USA) supplemented with 10% fetal bovine serum (FBS) (Gibco). Cells in exponential growth phase were stained with 200 nm MitoTracker Deep Red (Invitrogen, Grand Island, NY, USA) for 1 h and subsequently with 500 nm fluorescent TSPO ligand (Taliani *et al*., 2010) for 1.5 h while avoiding light bleaching. Before imaging, cells were fixed in 4% paraformaldehyde in phosphate-buffered saline (PBS) (137 mm NaCl, 2.7 mm KCl, 10 mm Na_2_HPO_4_, 2 mm KH_2_PO_4_, pH = 7.4). Photo-bleaching during imaging was minimized by mounting the cells in Vectashield medium.

### Longevity and oxidative stress/starvation/heat stress resistance assays

To monitor longevity on standard food, fruit flies were collected at 2–3 DAE by brief CO_2_ anesthesia and 20 flies placed in each vial with standard cornmeal agar medium. For female studies, only virgin flies were collected. Lifespan was determined at 25 °C and approximately 50% humidity with 12 h/12 h light/dark cycle. The number of dead flies was counted every 4 days and the surviving flies transferred to fresh cornmeal agar medium.

To monitor longevity in TSPO ligand-containing food, 5 μm to 50 μm PK11195 (1-(2-chlorophenyl)-N-methyl-N-(1-methylpropyl)isoquinoline-3-carboxamide) (Sigma, St. Louis, MO, USA) and 0.1 μm to 1 μm Ro5-4864 (4′-chlorodiazepam;7-chloro-5-(4-chlorophenyl)-1,3-dihydro-1-methyl-2H-1,4-benzodiazepin-2-one) (Sigma) were dissolved in distilled water. *Drosophila* at 3–5 DAE were starved for 3 h and transferred to vials containing filter papers soaked with drugs, diluted in 4 w/v% sucrose. Every 4–5 days, the number of dead flies was counted and the flies were transferred to a new drug-containing vial. Controls received filter paper with only 4% sucrose.

To monitor oxidative stress and starvation resistance, 3–6 DAE flies were maintained on standard cornmeal medium after eclosion and then transferred to vials containing filter papers previously soaked with 4 w/v% sucrose (control group), or 5 w/v% H_2_O_2_ in 4 w/v% sucrose (pH = 7.2) (oxidative stress group), or water without H_2_O_2_, sucrose (starvation group). The number of dead flies was recorded every few hours and the flies were transferred to new vials with new filter papers every 24 h. To monitor heat stress resistance, flies were maintained on standard cornmeal medium after eclosion and incubated in 37 °C. The number of dead flies was counted and the survival rate was calculated.

For longevity and oxidative stress assays, Kaplan–Meier statistics were used to determine the median lifespan/survival period, and log rank test was used to calculate *P* value to determine statistical significance. At least three independent measurements were performed for each experiment.

### Quantification of neurodegeneration

Fly heads were fixed in standard Bouin’s Fixative, embed in paraffin blocks, and sectioned. Sections were placed on slides and examined by fluorescent microscopy using the Rhodamine channel. The brain tissue fluoresced as red due to the endogenous fluorescence of the ‘white’ gene product. The appearance of nonfluorescent ‘black vacuoles’ within the brain indicated regions of neurodegeneration. To quantify neurodegeneration, the images of the sections were captured and the number of vacuoles counted.

### Western blotting

Ten *Drosophila* were homogenized in RIPA buffer (50 mm TrisHCl, 150 mm NaCl, 0.1% SDS, 1% Sodium deoxycholate, 1% Triton-X 100, 1 mm EDTA, pH 7.4) with protease inhibitor (Roche # 05892791001, Indianapolis, IN, USA) and the protein concentrations determined by the Bradford method (Bio-Rad, Hercules, CA, USA). Proteins, 40 μg per lane, were separated by 4–12% NuPAGE Bis-Tris gel (Invitrogen) and electroblotted on to PVDF membrane (Immobilin, Millipore, Billerica, MA, USA) at 200 mA for 15 h. The membranes were incubated overnight at 4 °C with rabbit anti-dTSPO polyclonal antibody (1:1000, Trevigen, Gaithersburg, MD, USA, or customized product from Covance), mouse anti-COX-I monoclonal antibody (1:1000, MitoScience, Eugene, OR, USA), mouse anti-VDAC monoclonal antibody (1:3000, Abcam, Cambridge, MA, USA), rabbit anti-β-actin monoclonal antibody (1:500, Santa Cruz Biotechnology AC-15, Dallas, TX, USA), or anti-α-tubulin monoclonal antibody (1:1000, Sigma). Membranes were washed four times with TBST (50 mm Tris, 150 mm NaCl, 0.05% Tween 20, pH 7.6), incubated with horseradish peroxidase-labeled goat anti-rabbit IgG (1:2000) for 1.5 h at room temperature, washed four times with TBST, incubated with the ECL protein blotting analysis system (Amersham, Pittsburgh, PA, USA) for 1 min and exposed to X-ray film for 2 min.

### Quantitative reverse transcriptase polymerase chain reaction

Total RNA was extracted from whole bodies of 20–40 flies or 100 fly heads using TRIZOL reagent (Invitrogen) followed by DNase treatment for whole body fly samples or RNeasy Mini Kit (Qiagen, Hilden, Germany) processing for fly heads. The RNA was converted to cDNA using oligo-d(T)15 (Invitrogen) and SuperScript II reverse transcriptase (Invitrogen). After reverse transcription, PCRs were performed using a 7500 Fast or ViiA7 Real-Time PCR System (Applied Biosystems, Grand Island, NY, USA), SYBR Green Master Mix (Applied Biosystems), and primers for Rp49 (forward, 5- gctaagctgtcgcacaaatg -3, and reverse, 5- ccaggaacttcttgaatccg -3) or dTSPO (forward, 5- ctcttcgtacccta cgtcgc -3, and reverse, 5- ctggttcgataggtcggaaa -3) or Aβ42 (forward, 5- cgcagttcctgagacttt -3, and reverse, 5- tatgacaacaccgcccac -3). The PCR protocol involved denaturation at 95 °C for 15 s and combined annealing and extension at 60 °C for 1 min over 40 cycles.

### Caspase 3/7 activity

Isolated 3rd-instar larval brain cells treated with H_2_O_2_, or isolated fly heads were homogenized firmly in Homogenization Buffer (225 mm mannitol, 75 mm sucrose, 10 mm MOPS, 1 mm EGTA, pH 7.2) on ice, then centrifuged at 300 g for 5 min. The supernatant was collected and an equal volume of reaction buffer (ApoONE kit, promega, Madison, WI, USA) was added in 96-well plate wells. The plate was shaken gently for 5 min, then incubated in dark for 15 h. Fluorescence was measured with fluorescent spectrophotometer (NOVOstar, BMG Labtech, Ortenberg, Germany), with the excitation at 499 nm and emission at 521 nm.

### γ-irradiation and TUNEL staining

The 3rd-instar larvae were subjected to γ-irradiation (30 Gray), allowed to recover for 3 h at room temperature, and their wing discs isolated for TUNEL staining. Wing discs were dissected and fixed for 20 min at room temperature in 4% PFA in PBS. The samples were then washed three times in PBT buffer (0.1% Tween-20 in PBS) for 10 min per wash, incubated in equilibration buffer (ApopTag kit; Millipore) for 1 h, and incubated again in reaction buffer (TdT enzyme; ratio 7:3; ApopTag kit) at 37 °C overnight. On the next day, the TdT reaction mix was replaced with stop buffer (diluted 1:34 in dH_2_O; ApopTag kit) and incubated at 37 °C for 3–4 h. The samples were washed three times, 5 min per wash, blocked in blocking solution (PBS, 0.3% Triton-X 100, and 5% normal goat serum) at room temperature for 1 h, and incubated with anti-digoxigenin antibody solution (diluted 47:53 in blocking solution; ApopTag kit) overnight in the dark at 4 °C. On the following day, the samples were washed four times in PBS, 20 min per wash, and imaged. Apoptosis was quantified by measuring the area of the TUNEL pixels and dividing it by the area of the total disc using ImageJ software (National Institutes of Health).

### Flow cytometry

Brains were dissected from 40–50 3rd-instar larvae flies and incubated in 1 × Trypsin-EDTA (GIBCO) at room temperature with gently agitated using 200 μL micropipette tips every 15–20 min until no visible tissue fragments could be seen. The cell suspension was centrifuged at 800 g for 5 min, the pellet washed twice with PBS, and resuspended with serum-free Schneider’s *Drosophila* Medium for apoptosis assay. After staining, the cell suspension was analyzed by Flow Cytometry (Accuri C6 Personal Flow Cytometer, BD Biosciences, San Jose, CA, USA).

Cell apoptosis was assessed by flow cytometry following Propidium Iodide (PI) /FITC-Annexin V double staining assay. FITC-Annexin V negative cells together with Propidium Iodide stained nuclei were excluded and dead versus healthy cells assessed by FITC-Annexin V staining. The cells were incubated with H_2_O_2_ for 3 h, and then spun down and washed with cold PBS. The pellet was resuspended with 1 × binding buffer (FITC-Annexin V Apoptosis Detection Kit I, BD Pharmingen, Franklin Lakes, NJ, USA). The density of the cells was adjusted to 1 × 10^6^ per mL and 100 μL of cell suspension was transferred to a 1.5-mL Eppendorf tube. The cells were then loaded with 5 μL FITC-Annexin V and 10 μL PI for 15 min at RT in the dark, 400 μL of Binding Buffer was added to each tube, and the relative fluorescence was assessed by flow cytometry.

### ATP content

Adenosine tri-phosphate content was determined by using an ATP Determination Kit (Molecular Probes, Grand Island, NY, USA). Five 7–10 DAE flies were homogenized on ice in 100 μL of water in 1.5-mL Eppendorf tube and the supernatant collected by centrifugation at 18 000 *g* for 5 min. This extract was diluted 1000-fold and 20 μL assayed in 96-well plates, the ATP content normalized to total protein.

### TBARS assay

Malondialdehyde content was determined by using the TBARS Assay Kit (Cayman Chemical, Ann Arbor, MI, USA). Twenty flies of 20–23 DAE were homogenized on ice in 250 μL of RIPA buffer in 1.5-mL Eppendorf tube and the supernatant collected by centrifugation at 1600 g for 10 min. This extract was assayed in 96-well plates without dilution. The MDA content was normalized to total protein.

### Mitochondrial biochemistry

#### Mitochondrial isolation

Unless otherwise indicated, mitochondria were isolated from 20–30, 2–5 DAE, whole flies. The flies were gently crushed in a 1.5-mL Eppendolf tube with 10 strokes of a fitted pestle in 1 mL Homogenization Buffer at 4 °C. The extracts were filtered through eight layers of cheesecloth and then centrifuged at 300 g for 5 min. The mitochondria were collected from the supernatant by centrifugation at 6000 g for 10 min. For respiration and membrane potential assays, the mitochondrial pellet was resuspended in 0.5 mL of Respiration Buffer (225 mm mannitol, 75 mm sucrose, 10 mm KCl, 10 mm TrisHCl and 5 mm KH_2_PO_4_, pH 7.2); For enzymatic assay of OXPHOS complexes, citrate synthase, and aconitase, the pellet was resuspended in 0.5 mL of hypotonic medium (25 mm K_2_HPO_4_, 5 mm MgCl2, pH 7.2) and the mitochondrial membranes disrupted by 2–3 liquid nitrogen freeze-thaw cycles.

#### Mitochondrial respiration

Respiration assays were performed in 1 mL of respiration buffer using a Clark-electrode. Oxygen consumption rates without (state IV) and with (state III) ADP were recorded (Tong *et al*., 2007). Respiration rates were normalized to Bradford protein content, corrected for BSA content.

#### Mitochondrial membrane potential

Mitochondrial membrane potential was measured by the mitochondrial uptake of 20 μm TMRM in a reaction containing 25 mm succinate. The decline in the buffer TMRM concentration was monitored at 575 nm with fluorescent spectrophotometer (NOVOstar, BMG Labtech). Relative membrane potentials were reported for tspo −/−, tspo +/− mitochondria relative to control +/+ mitochondria assayed in parallel wells.

#### OXPHOS complexes, citrate synthase, and aconitase assays

Oxidative phosphorylation complex assays were performed in 200 μL reactions in 96 well plate, monitored with a plate reader (SpectraMax Paradigm, Molecular Devices, Sunnyvale, CA, USA). The relative activities were normalized to the citrate synthase activity. Chemicals were from Sigma-Aldrich unless otherwise specified.

Complex I activity: Complex I (NADH:ubiquinone reductase) activity was determined by the rotenone-sensitive oxidation of NADH oxidation, monitored at 340 nm, using coenzyme Q analog decylbenzylquinone (DB) as an electron acceptor. The reaction buffer was (50 mm HEPES, 2.5 mg mL^−1^ BSA, 0.1 mm NADH, 10 mm KCN, 10 μg mL^−1^ antimycin A, 0.1 mm DB:H2, pH 7.2), with or without 10 μm rotenone.

Complex II activity: Complex II (ubiquinone succinate dehydrogenase) activity was determined by 2,6-dichlorophenolindophenol (DCPIP) oxidation at 600 nm coupled to the reduction of decylbenzylquinone in reaction solution (50 mm HEPES, 2.5 μm rotenone, 5 mm KCN, 2.5 μg mL^−1^ antimycin A, 20 mm sodium succinate, 50 μm DCPIP, 100 μm decylbenzylquinone, pH 7.2).

Complex III activity: Complex III (ubiquinol:cytochrome c reductase) activity was determined by the reduction of cytochrome c monitored at 550 nm, coupled to the oxidation of reduce decylbenzylquinol (DB:H_2_), in the reaction solution (50 mm HEPES, 2.5 mg mL^−1^ BSA, 1 mm DDM, 5 mm KCN, 10 μm rotenone, 0.2 μm reduced decylbenzylquinol, 50 μm cytochrome c/oxidized, pH 7.2).

Complex IV activity: Complex IV (cytochrome c oxidase) activity was determined by the oxidation of reduced cytochrome c, monitored at 550 nm in reaction solution (50 mm HEPES, 1 mm DDM, 50 μm cytochrome c/reduced, pH 7.2).

Complex V activity: Complex V (F1-ATP synthase) activity was measured as the rate of hydrolysis of ATP, generated by the conversion of phosphoenolpyruvate to pyruvate by pyruvate kinase (PK), linked to the reduction of pyruvate to lactate by lactate dehydrogenase (LDH). The reaction buffer was (40 mm TrisHCl, 20 μm EGTA, 0.2 mm NADH, 2.5 mm PEP, 25 μg mL^−1^ Antimycin A, 50 mm MgCl2, 0.5 mg mL^−1^ LDH, 0.5 mg mL^−1^ PK, 2.5 mm ATP, pH 8.0), with the rate being monitored by the oxidation of NADH at 340 nm. The reliance on proton pumping was confirmed by demonstrating the oligomycin (2 μm) sensitivity of the reaction.

Citrate synthase: Citrate synthase activity was determined as the reduction of 5, 5′-dithiobis-2-nitrobenzoic acid, monitored at 412 nm in reaction buffer (100 mm TrisHCl, 50 μm Acetyl-CoA, 0.1 mm DTNB, 0.1% Triton-X, 0.25 mm oxaloacetate, pH 7.4).

Aconitase activity and reactivation. Aconitase activity was assayed using the Aconitase Assay Kit (Cayman Chemistry Co.). Endogenous activity was determined in the initial extract and after reactivation with ferrous ammonium sulfate, the difference was used to indicate the extent of ROS inactivation. We used the percentage ratio of endogenous/reactivated total as the% aconitase inactivated by ROS.

## Supporting Information

**Fig. S1** Multiple sequences alignment of TSPO from various species aligned using CLUSTW. Amino acids on a black background are identical. Those on a gray background are similar for side chain hydrophobicity.

**Fig. S2** The duration of lifecycle in wild type and *tspo* −/− flies. The number of days from crossing to eclosion was recorded, for each genotype, *n* = 10 ~ 40. Each bar = mean ± SEM.

**Fig. S3** The sensitivity to starvation and heat stress in wild type and *tspo* −/− flies. (A) The male (upper) and female (lower) flies of 3-6 DAE were fed with water containing 4 w/v% sucrose (control group) or without sucrose (starvation group). Numbers of dead flies were counted and percent surviving calculated. *N* = 10–30 flies for each curve. (B) The male 3–6 DAE flies fed with regular food were incubated in 37 °C, the rate of survival was monitored after 15-h incubation. *N* = 6 for each group, indicating six vials of flies.

**Fig. S4** The up-regulation of dTSPO expression during aging. (A) Levels of dTSPO mRNA (determined by qRT-PCR, Rp49 mRNA reference) and (B) levels of protein (assessed by densitometry of western blot, β-actin reference). In both experiments, *tspo* +/+ male flies were maintained on standard cornmeal medium at 22 °C. For mRNA at DAE 0 (*n* = 8), 25 (*n* = 8), 50 (*n* = 6), 75 (*n* = 3). For protein, *n* = 2 at each time point.

**Fig. S5** Effect of dTSPO depletion in neurons on Aβ42 mRNA expression level. Total mRNA was extracted from heads of 1–3 DAE male Aβ42-expressing flies with or without neuronal knockdown of dTSPO. The dTSPO mRNA level was monitored by qRT-PCR. (*n* = 3 for each genotype)

**Fig. S6** Effect of dTSPO genetic depletion on *Drosophila* OXPHOS complex activity and ROS production at 2–4 DAE. Mitochondria were isolated from whole body lysates of *tspo* +/+, +/−, and −/− flies (mixture of equal number of male and female). (A) Mitochondrial oxygen consumption rates were measured while metabolizing site I (pyuvate + malate) substrates in the absence (state 4) or presence (state 3) of ADP, *N* = 8 for each genotype. (B) Relative mitochondrial membrane potential assessed by TMRM uptake in mitochondria, *N* = 3 per genotype. (C) Mitochondrial OXPHOS complex I-IV activities normalized to citrate synthase activity, *N* = 4 repetitions per assay. (D) Mitochondrial aconitase activity in male and female *tspo* +/+ and −/− flies, expressed as the ratio of endogenous activity divided by total activity following aconitase following reactivation with Fe^2+^. *N* = 3 for each genotype. For all data above, **P* < 0.05, ***P* < 0.01, ****P* < 0.001.

**Fig. S7** Effect of dTSPO depletion on ATP content. ATP content in whole body tissue of 7–10 DAE flies was similar in *tspo* −/− flies compared with *tspo* +/+ wild type controls. For each genotype, *n* = 3.

**Fig. S8** Effect of dTSPO depletion on MDA level in TBARS assay. MDA level in whole body tissue of 20–23 DAE flies was similar in *tspo* −/− flies compared with *tspo* +/+ wild-type controls. For each genotype, *n* = 3.

**Table S1.** Median, maximum lifespan and statistical analysis of dTSPO mutant, knockdown flies and wild-type flies treated with PK11195 and Ro5-4864. **Table S2.** Median, maximum lifespan and statistical analysis of Aβ42-expressing flies with modification of dTSPO inactivation.
