# Supplementary figures and images for: Genetic analysis of dTSPO, an outer mitochondrial membrane protein, reveals its functions in apoptosis, longevity, and Aβ42-induced neurodegeneration
Source: Aging Cell. 2014 Feb 21;13(3):507–18. doi: 10.1111/acel.12200 (PMC4076708; doi:10.1111/acel.12200)

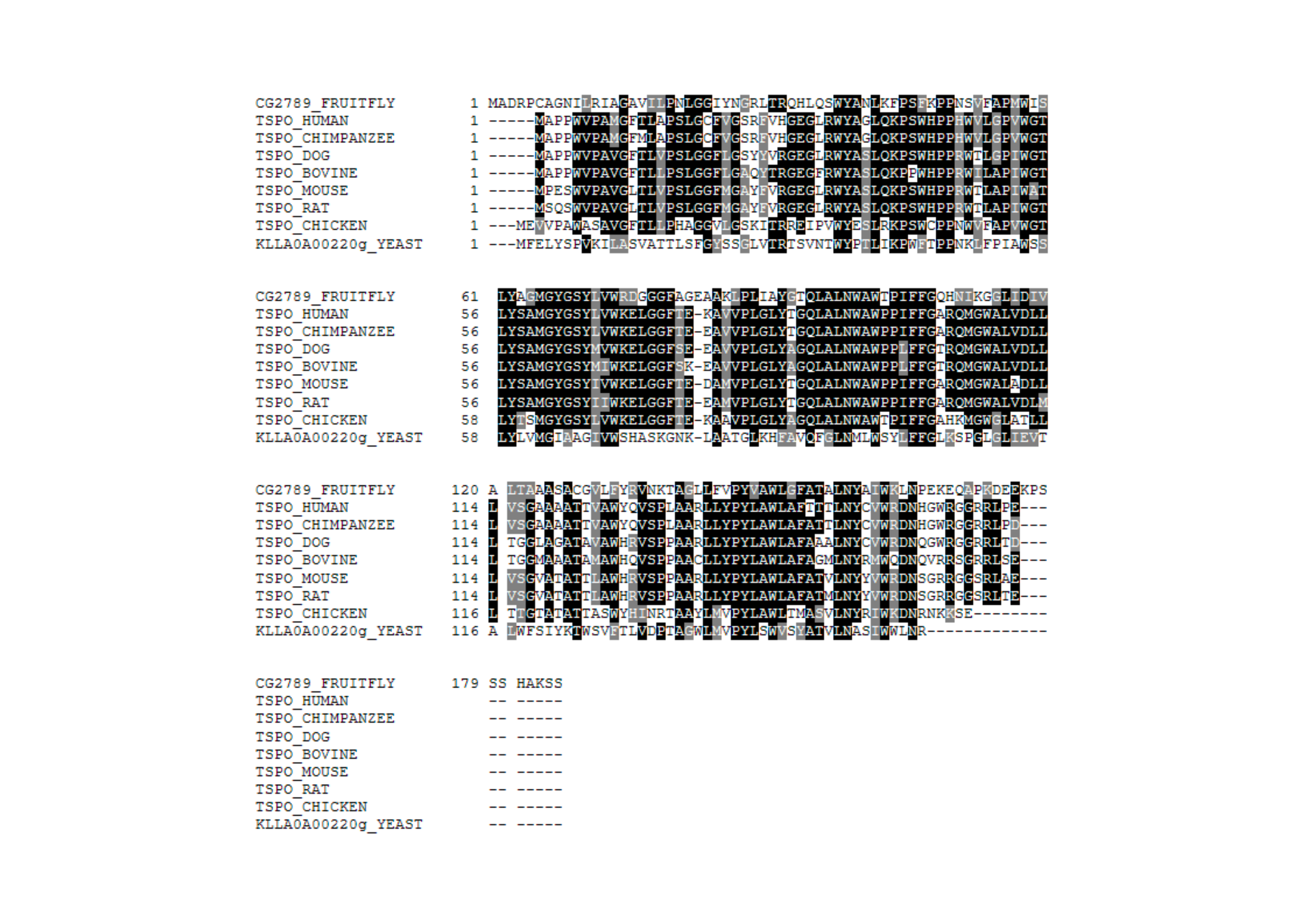

Supplement: Supplementary file 1 — Fig. S1 Multiple sequences alignment of TSPO from various species aligned using CLUSTW. Amino acids on a black background are identical. Those on a gray background are similar for side chain hydrophobicity. [file acel0013-0507-sd1.tiff]

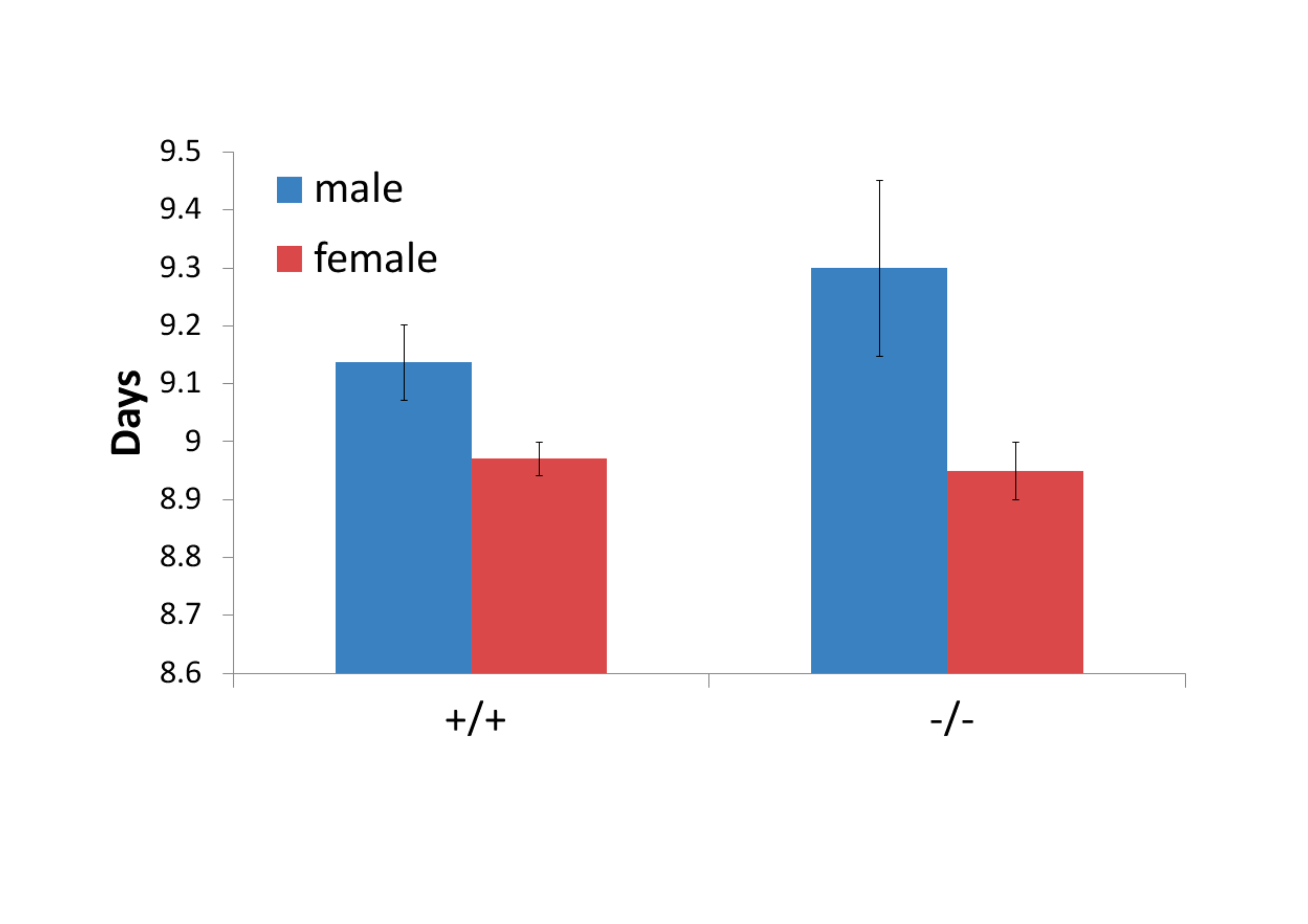

Supplement: Supplementary file 2 — Fig. S2 The duration of lifecycle in wild type and tspo −/− flies. The number of days from crossing to eclosion was recorded, for each genotype, n = 10 ~ 40. Each bar = mean ± SEM. [file acel0013-0507-sd2.tiff]

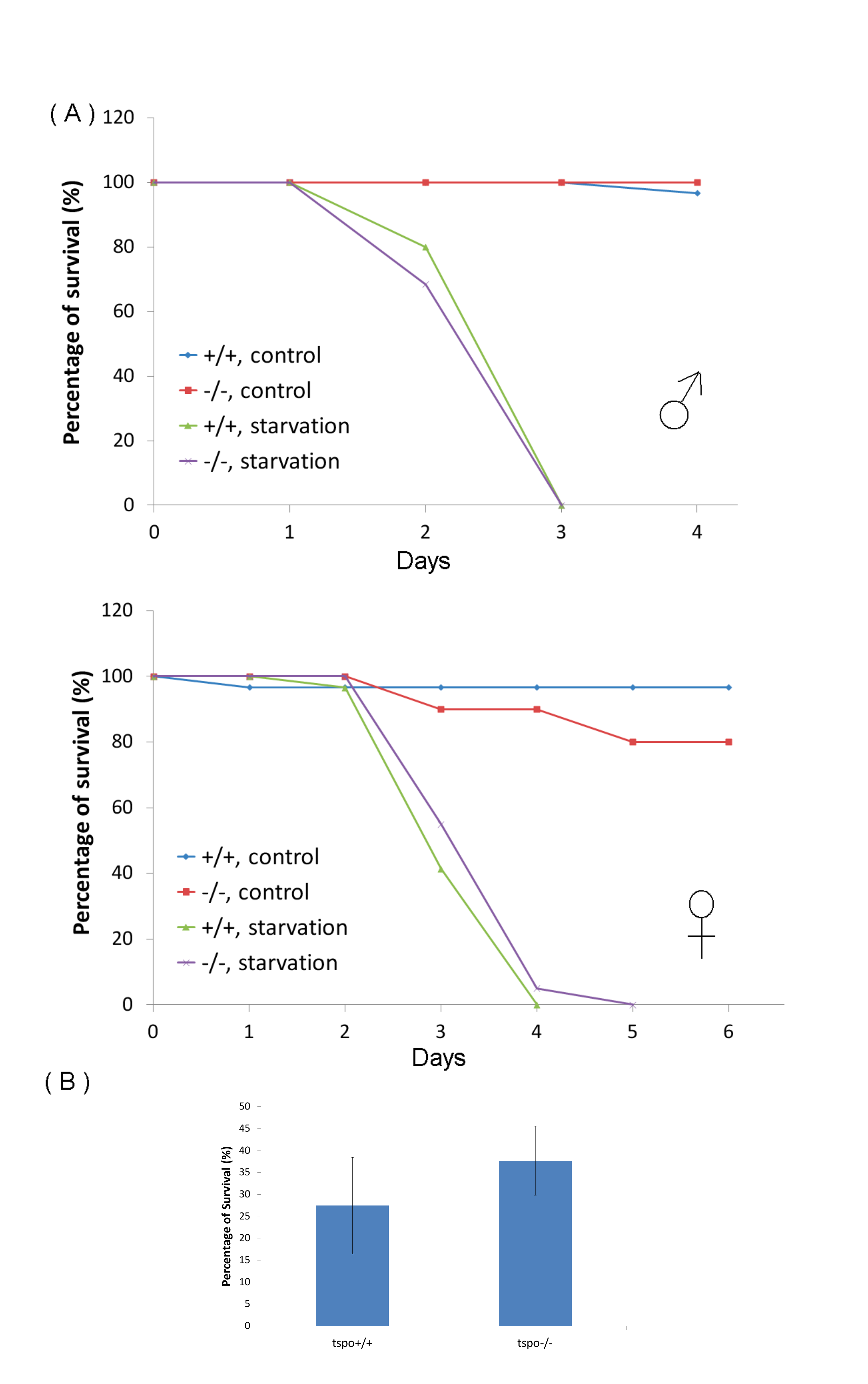

Supplement: Supplementary file 3 — Fig. S3 The sensitivity to starvation and heat stress in wild type and tspo −/− flies. (A) The male (upper) and female (lower) flies of 3-6 DAE were fed with water containing 4 w/v% sucrose (control group) or without sucrose (starvation group). Numbers of dead flies were counted and percent surviving calculated. N = 10–30 flies for each curve. (B) The male 3–6 DAE flies fed with regular food were incubated in 37 °C, the rate of survival was monitored after 15-h incubation. N = 6 for each group, indicating six vials of flies. [file acel0013-0507-sd3.tiff]

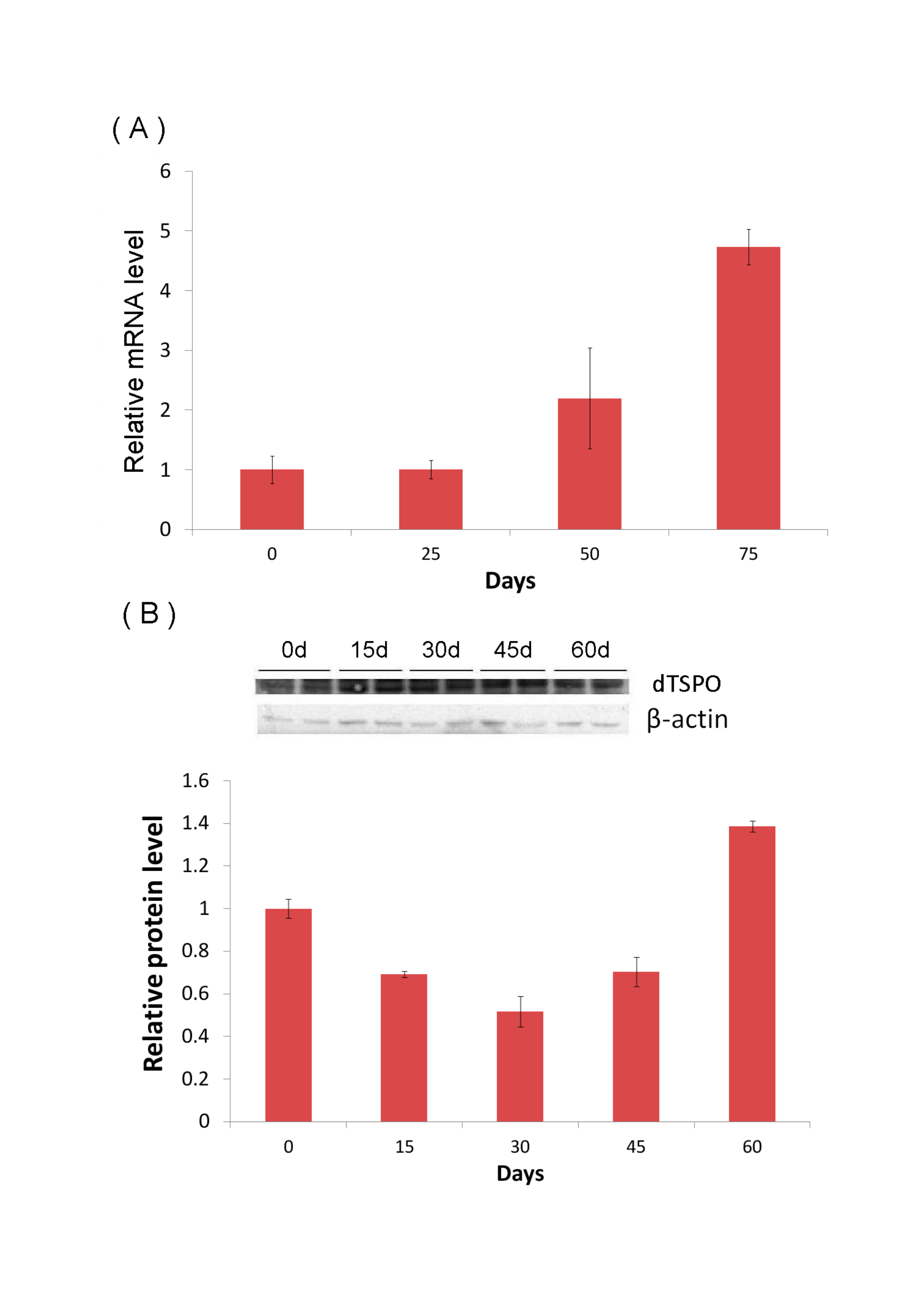

Supplement: Supplementary file 4 — Fig. S4 The up-regulation of dTSPO expression during aging. (A) Levels of dTSPO mRNA (determined by qRT-PCR, Rp49 mRNA reference) and (B) levels of protein (assessed by densitometry of western blot, β-actin reference). In both experiments, tspo +/+ male flies were maintained on standard cornmeal medium at 22 °C. For mRNA at DAE 0 (n = 8), 25 (n = 8), 50 (n = 6), 75 (n = 3). For protein, n = 2 at each time point. [file acel0013-0507-sd4.tiff]

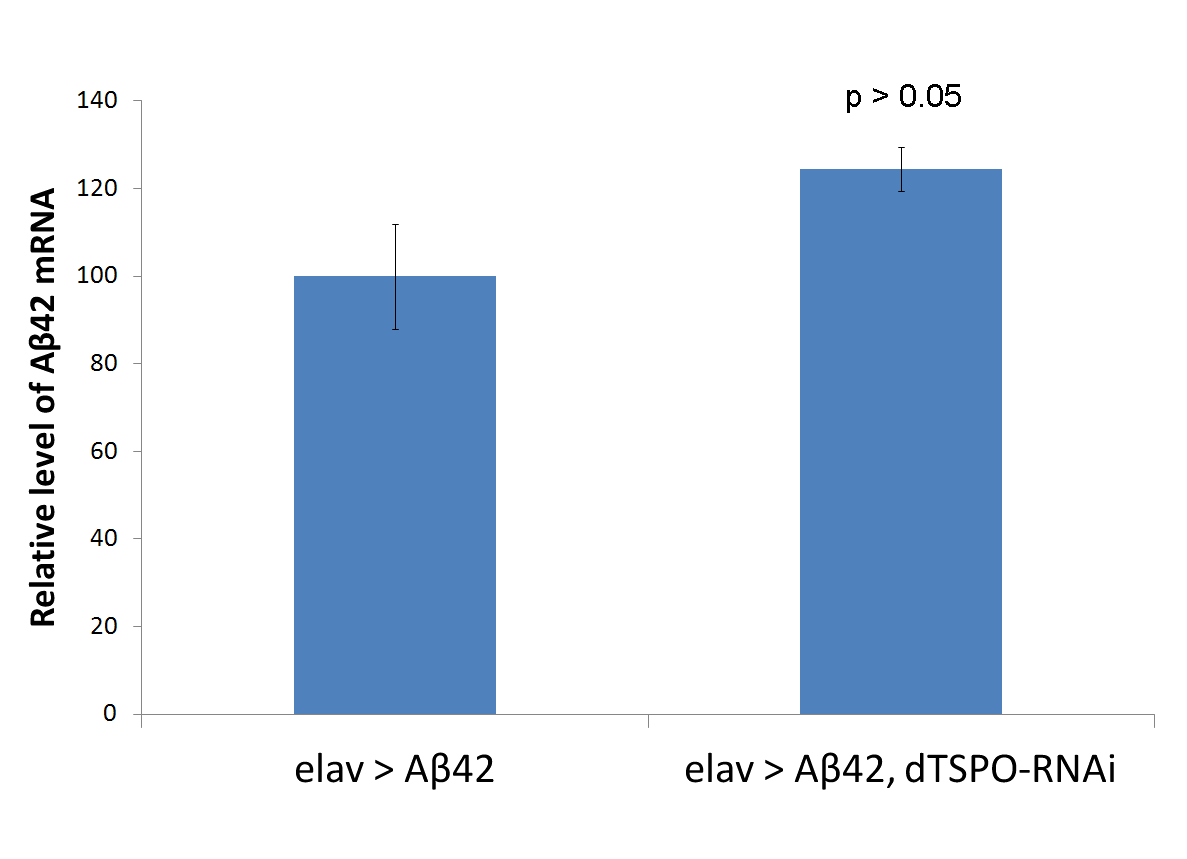

Supplement: Supplementary file 5 — Fig. S5 Effect of dTSPO depletion in neurons on Aβ42 mRNA expression level. Total mRNA was extracted from heads of 1–3 DAE male Aβ42-expressing flies with or without neuronal knockdown of dTSPO. The dTSPO mRNA level was monitored by qRT-PCR. (n = 3 for each genotype) [file acel0013-0507-sd5.tif]

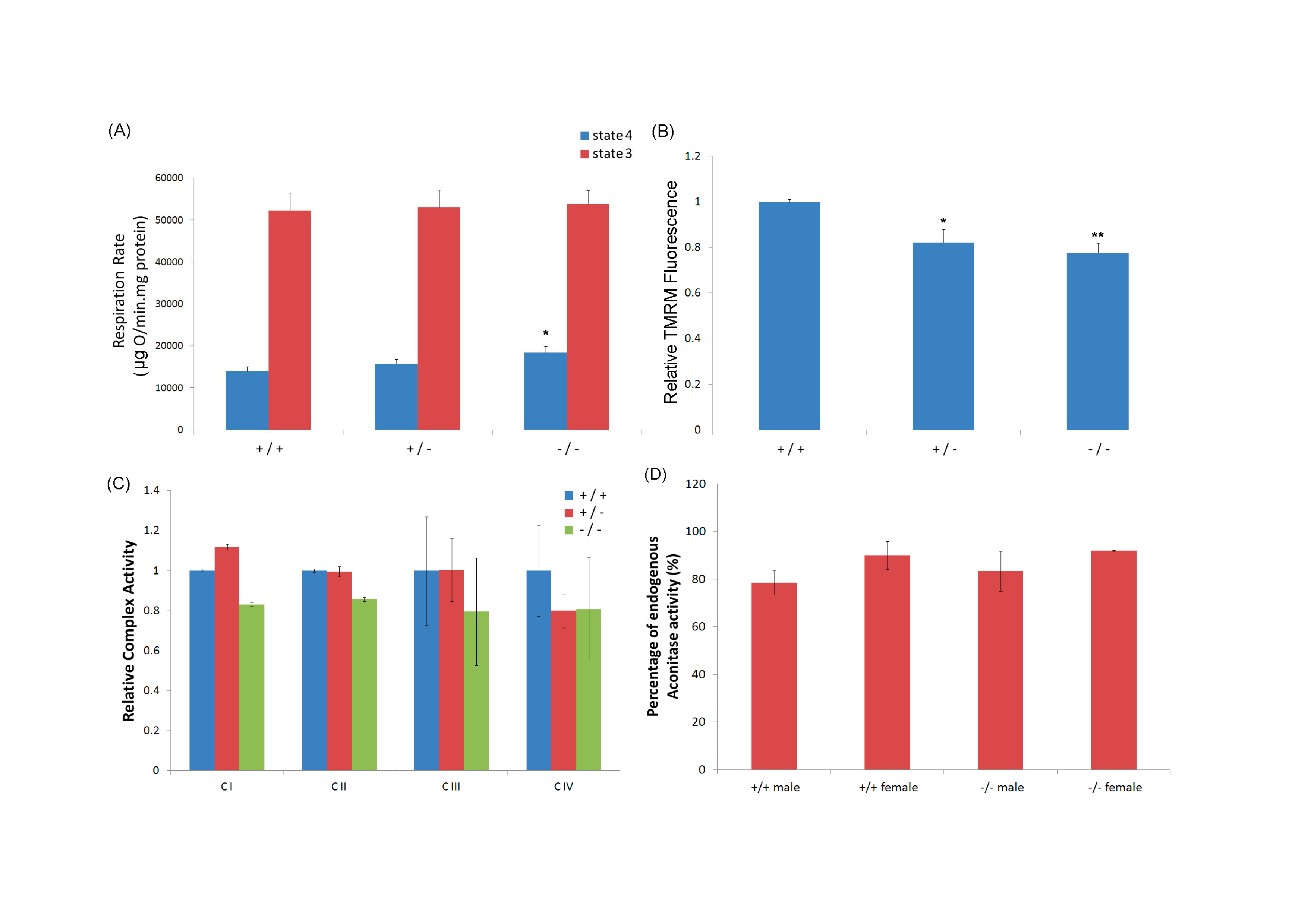

Supplement: Supplementary file 6 — Fig. S6 Effect of dTSPO genetic depletion on Drosophila OXPHOS complex activity and ROS production at 2–4 DAE. Mitochondria were isolated from whole body lysates of tspo +/+, +/−, and −/− flies (mixture of equal number of male and female). (A) Mitochondrial oxygen consumption rates were measured while metabolizing site I (pyuvate + malate) substrates in the absence (state 4) or presence (state 3) of ADP, N = 8 for each genotype. (B) Relative mitochondrial membrane potential assessed by TMRM uptake in mitochondria, N = 3 per genotype. (C) Mitochondrial OXPHOS complex I-IV activities normalized to citrate synthase activity, N = 4 repetitions per assay. (D) Mitochondrial aconitase activity in male and female tspo +/+ and −/− flies, expressed as the ratio of endogenous activity divided by total activity following aconitase following reactivation with Fe2+. N = 3 for each genotype. For all data above, *P < 0.05, **P < 0.01, ***P < 0.001. [file acel0013-0507-sd6.tiff]

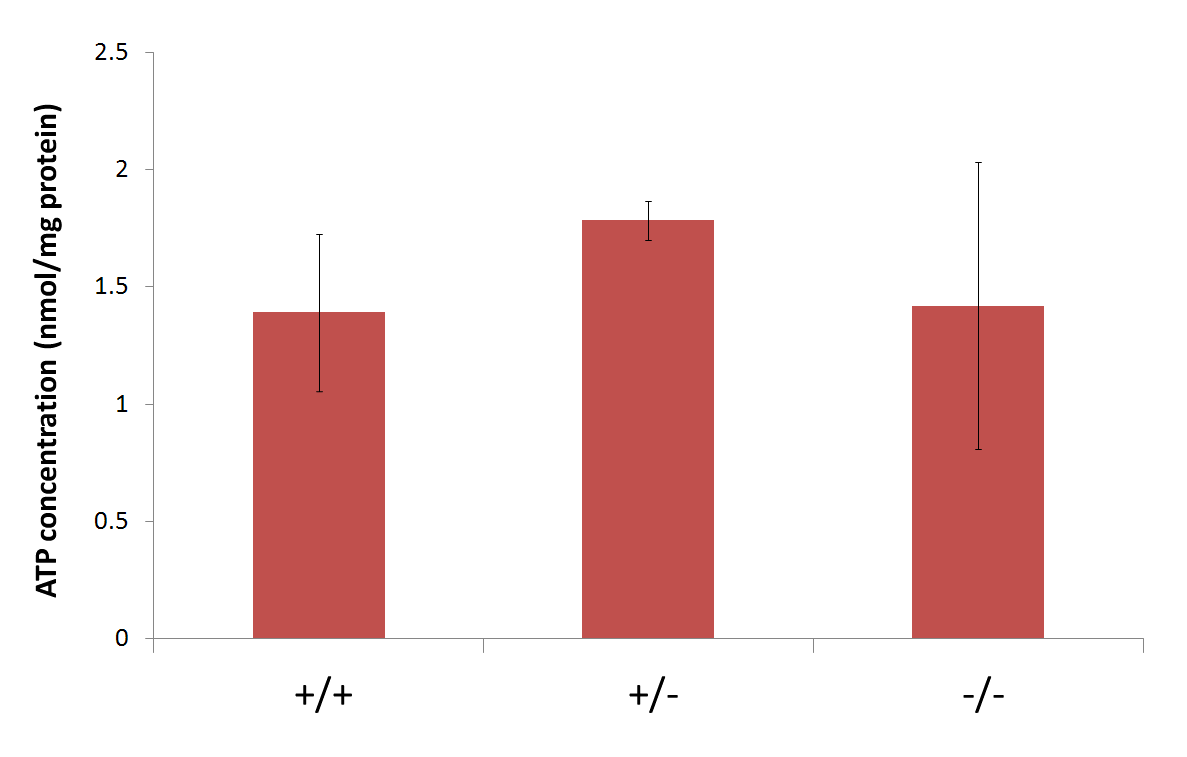

Supplement: Supplementary file 7 — Fig. S7 Effect of dTSPO depletion on ATP content. ATP content in whole body tissue of 7–10 DAE flies was similar in tspo −/− flies compared with tspo +/+ wild type controls. For each genotype, n = 3. [file acel0013-0507-sd7.tif]

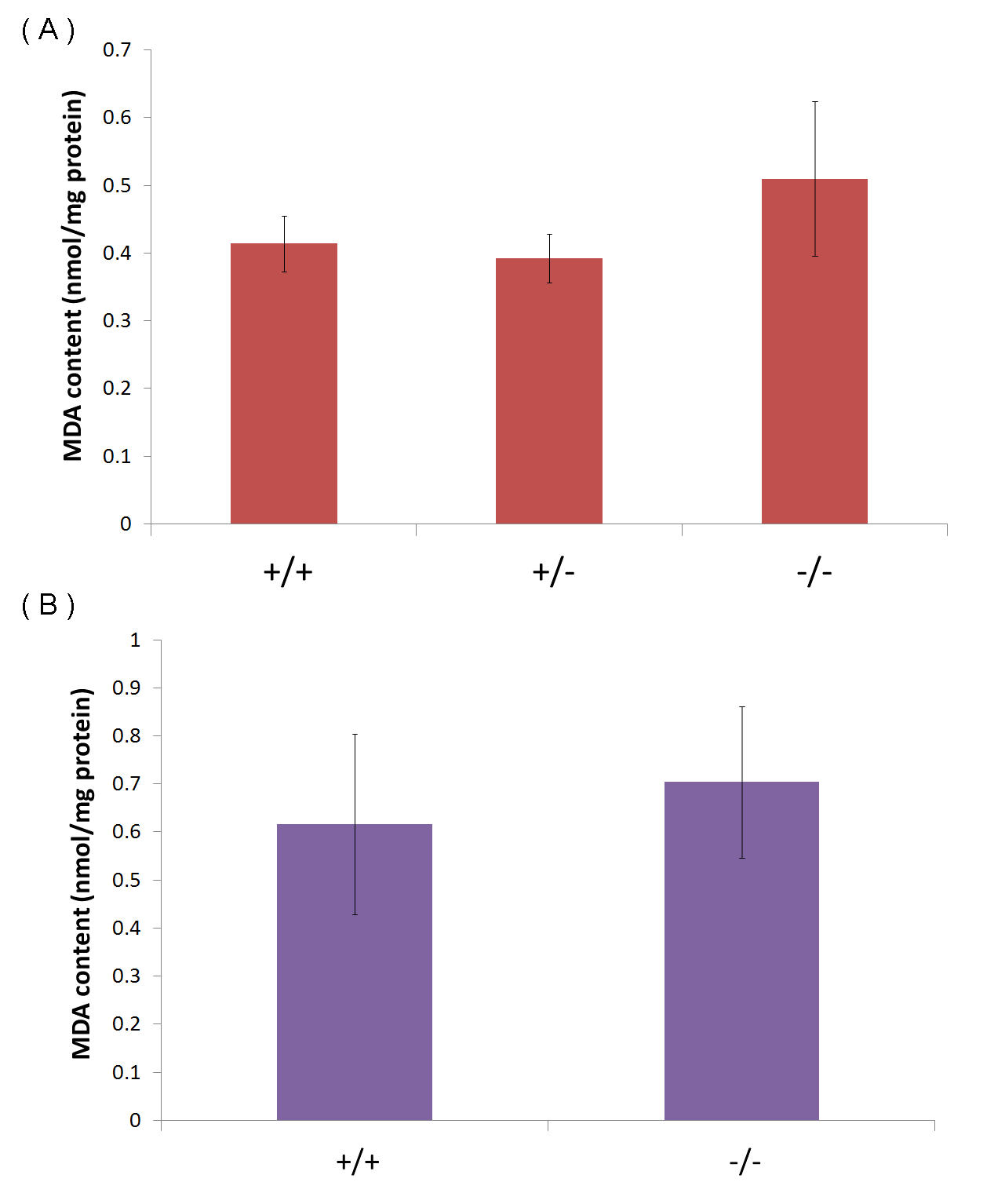

Supplement: Supplementary file 8 — Fig. S8 Effect of dTSPO depletion on MDA level in TBARS assay. MDA level in whole body tissue of 20–23 DAE flies was similar in tspo −/− flies compared with tspo +/+ wild-type controls. For each genotype, n = 3. [file acel0013-0507-sd8.tif]
