# Supplementary material for: Genetic analysis of dTSPO, an outer mitochondrial membrane protein, reveals its functions in apoptosis, longevity, and Aβ42-induced neurodegeneration
Source: Aging Cell. 2014 Feb 21;13(3):507–18. doi: 10.1111/acel.12200 (PMC4076708; doi:10.1111/acel.12200)
Supplement: Supplementary file 9 — Table S1. Median, maximum lifespan and statistical analysis of dTSPO mutant, knockdown flies and wild-type flies treated with PK11195 and Ro5-4864. Table S2. Median, maximum lifespan and statistical analysis of Aβ42-expressing flies with modification of dTSPO inactivation. [file acel0013-0507-sd9.doc]

Table S1. Median, maximum lifespans and statistical analysis of dTSPO mutant, knock-down flies and wild type flies treated with PK11195 and Ro5-4864.

|  | Number of flies recorded | median lifespan (days) | maximum lifespan (days) | P value |
| --- | --- | --- | --- | --- |
|
|  |  |  |  |  |
| control for PK11195 | 86 | 23.3 | 39 |  |
| 5μM PK11195 | 78 | 29.5 | 45 | 0.0001 (Vs control) |
| 50μM PK11195 | 88 | 22.98 | 42 | 0.5187 (Vs control) |
|  |  |  |  |  |
| control for Ro5-4864 | 62 | 18.87 | 39 |  |
| 0.1μM Ro5-4864 | 64 | 21 | 39 | 0.2363 (Vs control) |
| 1μM Ro5-4864 | 72 | 17.71 | 39 | 0.9448 (Vs control) |
|  |  |  |  |  |
| tspo+/+ male | 273 | 43.74 | 64 |  |
| tspo-/- male | 349 | 52.28 | 72 | <0.0001 |
|  |  |  |  |  |
| tspo+/+ female | 288 | 54.97 | 88 |  |
| tspo-/- female | 331 | 53.38 | 92 | 0.8468 |
|  |  |  |  |  |
| Dcr2; actGal4/+ male | 94 | 51.7 | 64 |  |
| Dcr2; actGal4/dTSPO-RNAi male | 54 | 58.44 | 84 | <0.0001 |
|  |  |  |  |  |
| Dcr2; actGal4/+ female | 63 | 68.76 | 88 |  |
| Dcr2; actGal4/dTSPO-RNAi female | 94 | 70.55 | 96 | 0.0117 |

**Table S2. Median, maximum lifespans and statistical analysis of Aβ42-expressing flies with modification of dTSPO inactivation.**

|  | Number of flies recorded | median lifespan (days) | maximum lifespan (days) | P value |
| --- | --- | --- | --- | --- |
|
| Male |  |  |  |  |
| elav Gal4 control | 295 | 59.16 | 76 |  |
| elav > Aβ42 | 108 | 53.33 | 76 | <0.0001 (Vs elav Gal4 control) |
| elav>Aβ42, tspo+/- | 190 | 57.85 | 72 | 0.0006 (Vs elav>Aβ42) |
| elav>Aβ42, dTSPO-RNAi | 163 | 62.5 | 80 | <0.0001 (Vs elav>Aβ42) |
| elav>dTSPO-RNAi | 266 | 56.08 | 80 |  |
|  |  |  |  |  |
| Female |  |  |  |  |
| elav Gal4 control | 139 | 59.8 | 84 |  |
| elav > Aβ42 | 93 | 54.88 | 84 | <0.0001 (Vs elav Gal4 control) |
| elav>Aβ42, tspo+/- | 87 | 61.1 | 76 | 0.0001 (Vs elav>Aβ42) |
| elav>Aβ42, dTSPO-RNAi | 77 | 58.7 | 72 | 0.0209 (Vs elav>Aβ42) |
| elav>dTSPO-RNAi | 234 | 65.49 | 80 |  |
